# Protective effect of ligustrazine on oxidative stress and apoptosis following testicular torsion in rats

**DOI:** 10.1038/s41598-023-47210-9

**Published:** 2023-11-21

**Authors:** Songmao Chen, Zhengjian Liao, Tingting Zheng, Yuanfan Zhu, Liefu Ye

**Affiliations:** 1 Provincial Clinical Medical College of Fujian Medical University, Fuzhou, 350001 Fujian China; 2 Department of Urology, Fujian Provincial Hospital, Fuzhou, 350001 Fujian China; 3 Department of Neurosurgery, Fujian Provincial Hospital, Fuzhou, 350001 Fujian China; 4 Fujian Medical University, Fuzhou, 350122 Fujian China

**Keywords:** Diseases, Reproductive disorders, Urogenital diseases

## Abstract

Testicular torsion is a common urologic emergency and one of the causes of infertility in males. It has been reported that ligustrazine may decrease oxidative stress and reduce ischemia–reperfusion injury. This study aims to investigate the protective effect of ligustrazine in ischemia–reperfusion injury after testicular torsion-detorsion. First, 40 rats were randomly and equally divided into TMP (Ligustrazine) group, the Testicular torsion (T/D) group, the Sham (Sham operation) group, and Control group. The left testis of rats in the TMP and T/D group was rotated for 2 h. The TMP group was intraperitoneally injected with ligustrazine solution and the T/D and the Sham groups were injected with normal saline. The left testes of four groups were obtained for assay on the 4th day after the operation. Average level of superoxide dismutase (SOD), glutathione peroxidase (GPX), and catalase (CAT) were higher in Sham and Control groups than T/D group and TMP group. Conversely, average level of malondialdehyde (MDA) and reactive oxygen species (ROS) was lower in Sham and Control groups than T/D group and TMP group. In contrast with the T/D group, SOD, GPX, and CAT enzymatic activities increased, whereas MDA and ROS content decreased in the TMP group (*P *< 0.05). Microscopic observation showed that the testicular tissue of the Sham and Control groups were basically normal. The TMP and T/D groups had significant testicular tissue damage, whereas the TMP group had less damage and apoptosis than the T/D group. The apoptotic index of germ cells in the TMP group (13.05 ± 4.41) was lower than the T/D group (30.23 ± 11.31) (*P *< 0.05) and higher (*P *< 0.05) than the Sham group (0.56 ± 0.29). So we found that Ligustrazine lowered ischemia–reperfusion injury after testicular torsion-detorsion by decreasing the reactive oxygen species and suppressing apoptosis.

## Introduction

Testicular torsion is a typical urological emergency that occurs among adolescent males. It has an incidence of approximately 4 in 10,000 men younger than 25 years and accounts for about 13–54% of emergency scrotum diseases in children^1–3^. Testicular torsion manifests as scrotal pain, and lower abdominal pain in a few cases; it can easily be misdiagnosed as epididymal-orchitis, varicocele, or even appendicitis. Blood is supplied to the testis through the terminal artery, and there is insufficient collateral circulation in this condition. Due to the twisted spermatic cord compressing the testicular artery, testicular torsion induces a loss of blood supply to the testis. Thus, immediate diagnosis and surgery of torsion prevent necrosis of the torqued testis. The resuscitated testis has a salvage rate of 90% if the torsion time is less than 6 h, 50% if the torsion time is less than 12 h, and less than 10% if the torsion time is more than 24 hours^4^. Nonetheless, testicular atrophy and diminished fertility might still occur to varying degrees after resuscitation. Hence, exploring therapeutic approaches to minimize testicular injury due to testicular torsion is a hot component of current research. In this study, we aimed to explore effective therapeutic agents.

Ischemia–reperfusion injury (IRI) after testicular torsion is the primary pathophysiological mechanism that causes testicular injury^1^. Testicular ischemia due to testicular torsion produces excessive production of reactive oxygen species (ROS), which can lead to oxidative stress (OS) if the ischemia lasts too long resulting in insufficient or ineffective cellular antioxidant capacity to counteract ROS formation^5^. Damage to spermatozoa by OS can manifest as insufficient energy metabolism, lipid peroxidation and DNA damage, resulting to loss of motility and viability^6^. Ligustrazine, otherwise known as tetramethylpyrazine (TMP), is an important element of Chuanxiong, a traditional Chinese medicine. It has proven effective in decreasing ischemia–reperfusion injury (IRI) in various organs, including the brain, heart, kidneys, and liver, due to its antioxidant, anti-inflammatory, and antiplatelet effects^7–10^. Yang DP's research has revealed that TMP can improve monocrotaline-Induced pulmonary hypertension through the ROS/iNOS/PKG pathway. The mechanisms underlying this may be linked to its anti-inflammatory, antioxidant, and antiproliferative properties in pulmonary arter^11^. Recent study found that TMP can significantly reduce the formation of NETs and inhibit the production of ROS in neutrophils, thus alleviating hepatic IRI^10^. In another study, TMP was found to have strong antioxidant activity, inhibiting reactive oxygen species generation and oxidative stress, thereby reducing iron overload-induced apoptosis in vascular endothelial cells^12^. At present, treatment of testicular torsion-detorsion with ligustrazine is not studied, Therefore, for the first time, this work assessed the protective effect and mechanism of ligustrazine on IRI after testicular torsion-detorsion.

## Materials and methods

### Animals

Experimental protocols were approved by the ethics committee of Fujian Medical University (IACUC FJMU 2022-0433) prior to starting and strictly followed to ensure animal welfare. all methods were carried out in accordance with relevant guidelines and regulations. All methods were carried out in accordance with ARRIVE guidelines and regulations (https://arriveguidelines.org). 40 healthy male Sprague Dawley rats were purchased from Wu Laboratory Animal Company (Certificate of Conformity No. 2008001644108), weighing 350–400 g. The 12-h light/dark cycle was performed in a temperature-controlled room (22 ± 2 °C and 55 ± 5% humidity) with free access to standard animal food and water. All experiments were conducted between 10:00 am and 3:00 pm.

### Chemicals

Ligustrazine and DMSO were provided by the Yuanye Bio-Technology (Shanghai, China). Detection kits (*GPX*, *SOD*, *CAT*, *MDA, ROS*) were purchased from Solarbio (Beijing, China), and Paraformaldehyde Fixative was procured from Biosharp (Hefei, China). Further, 0.9% normal saline was bought from Haiwang Fuyao Pharmaceutical (Fuzhou, China), and pentobarbital sodium was purchased from Sigma (Shanghai, China). Ligustrazine was freshly prepared by dissolving in 4% DMSO and 0.9% normal saline.

### Surgical procedure and experimental design

**Figure 1:**
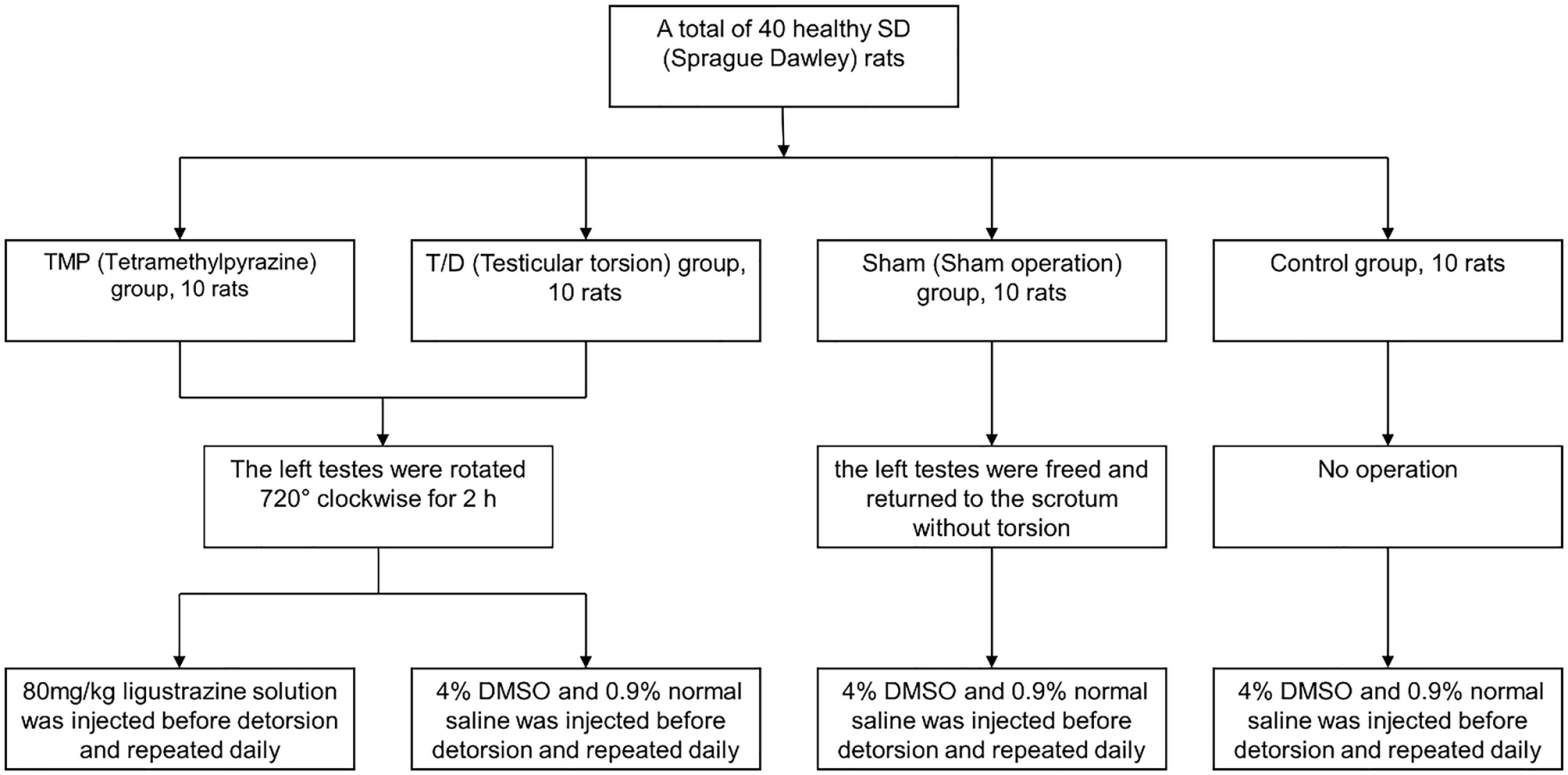
The experimental design and surgical procedure.

A total of 40 healthy 8–10 weeks old SD (Sprague Dawley) rats were divided into TMP (Ligustrazine) group, T/D (Testicular torsion) group, Sham (Sham operation) group, and Control group, with ten rats in each group with randomization methods. As show in Fig. 1, the testicular torsion model was established using Turner’s method^13^: A small incision was made in the left lower abdomen of rats in the TMP and T/D groups, and the left testes were freed. The left testes were rotated 720° clockwise. The incision was closed after fixing the twisted testis with a silk thread to prevent automatic detorsion. After 2 h, the original incision was opened, the left testis was repositioned, and the incision was sutured. The rats of Control group were kept without surgery or injection of any drugs. In the Sham group, a sham operation was performed, where the left testis was freed and returned to the scrotum without torsion before closing the incision. The TMP group was intraperitoneally injected with 80 mg/kg ligustrazine solution 30 min before detorsion (The dose of TMP was developed based on relevant papers and our preliminary experiments^14,15^) and repeated daily after the operation at 3:00 PM for 3 days. The T/D and the Sham groups were intraperitoneally injected with a volume similar to 4% DMSO and 0.9% normal saline. All animals were anesthetized through intraperitoneal injection of 2% pentobarbital sodium (35 mg/kg).

### Specimen analysis

All the rats were euthanized at 3:00 pm on the 4th day after the operation, and the left testes of four groups were obtained for analysis. Each testis was subdivided into two equal parts; one part was immediately fixed in 4% paraformaldehyde for 24 h and then embedded in paraffin. The remaining part was chopped using ophthalmic scissors and mixed with 9 times the weight of normal cold saline before being homogenized in a tissue homogenizer. The homogenate was placed in a 4 °C centrifuge and centrifuged at 223.6 g for 10 min. The activities of superoxide dismutase (SOD), glutathione peroxidase (GPX), catalase (CAT), and content of malondialdehyde (MDA) in the supernatant of each sample were detected by using activity assay kits (*GPX, SOD, CAT, MDA*) according to the instruction procedure, respectively. Additionally, The content of reactive oxygen species (ROS) was detected by fluorescent probe DCFH-DA in strict accordance with the instructions of the assay kit.

### Histopathologic evaluation and TUNEL

The testicular tissue was cut into paraffin sections and divided into two parts. One part was stained with hematoxylin–eosin (HE) and the damage to the testicular tissue was observed under a microscope by an experienced pathologist. A total of 10 roundest seminiferous tubules were selected from each section and scored using the Johnsen 10-level scoring method^16^. Another part of the paraffin sections was examined for spermatogenic cell apoptosis in the tissue sections using the in situ terminal deoxynucleotidyl transferase-mediated deoxyuridine tripho-sphate–biotin nick end labeling (TUNEL) method^17^, and 10 roundest spermatogonial tubules were randomly selected from each tissue section for observation, and intraductal apoptotic spermatogenic cells were enumerated and averaged.

### Statistical analysis

Sample size calculation in this study used Resource Equation Approach^18^. Statistical analyses were performed using the GraphPad Prism 8.0 software (San Diego, CA, USA). All the data were pre-verified by K-S test and analyzed by applying the Levene Test. Student T-test would be employed if conditions were met, in which the measurement data of normal distribution were expressed as mean ± SD, while that of skewed distribution were analyzed by Mann–Whitney U test and indicated by the median and interquartile range. Differences with a *P* value of less than 0.05 (*P *< 0.05) were considered statistically significant.

### Ethical approval

The protocol of this study was approved by the ethics committee of Fujian Medical University (IACUC FJMU 2022-0433). The study is also reported in accordance with ARRIVE guidelines.

## Results

### SOD, GPX, CAT activity determination, and MDA, ROS content determination

Compared to the Sham and Control groups, enzymatic activities of *SOD, GPX*, and *CAT* were observed to decrease, while *MDA* and *ROS* content increased in the TMP and T/D groups. These differences exhibited statistical significance (*P *< 0.05). In contrast to the T/D group, the TMP group demonstrated significant increases in *SOD, GPX*, and *CAT* enzymatic activities along with decreased *MDA* and *ROS* content. These differences were also found to be statistically significant (*P *< 0.05) (refer to Table 1). Therefore, Ligustrazine could alleviate oxidative stress in the testis.

**Table 1:** Comparison of *SOD*, *GPX*, *CAT* activities, and *MDA, ROS* content in the testis of different groups of rats (x ± s).

Group	n	ROS (U/mg prot)	SOD (U/mg prot)	MDA (nmol/mg prot)	GPX (U/mgprot)	CAT (U/mg prot)
TMP	10	0.69 ± 0.11 abc	49.67 ± 4.59 abc	2.66 ± 0.04 abc	28.46 ± 1.58 abc	266.58 ± 7.28 abc
T/D	10	1.05 ± 0.19 bc	28.04 ± 3.56 bc	4.61 ± 0.20 bc	19.25 ± 1.03 bc	156.89 ± 9.56 bc
Sham	10	0.19 ± 0.08	63.67 ± 3.39	0.88 ± 0.11	112.23 ± 1.36	367.24 ± 5.03
Control	10	0.25 ± 0.09	68.50 ± 2.50	0.92 ± 0.08	113.00 ± 1.10	368.35 ± 6.55

(a) *P *< 0.05 versus the T/D group; (b) *P *< 0.05 versus the Sham group; (c) *P *< 0.05 versus the Control group.

### Histomorphological changes

**Figure 2:**
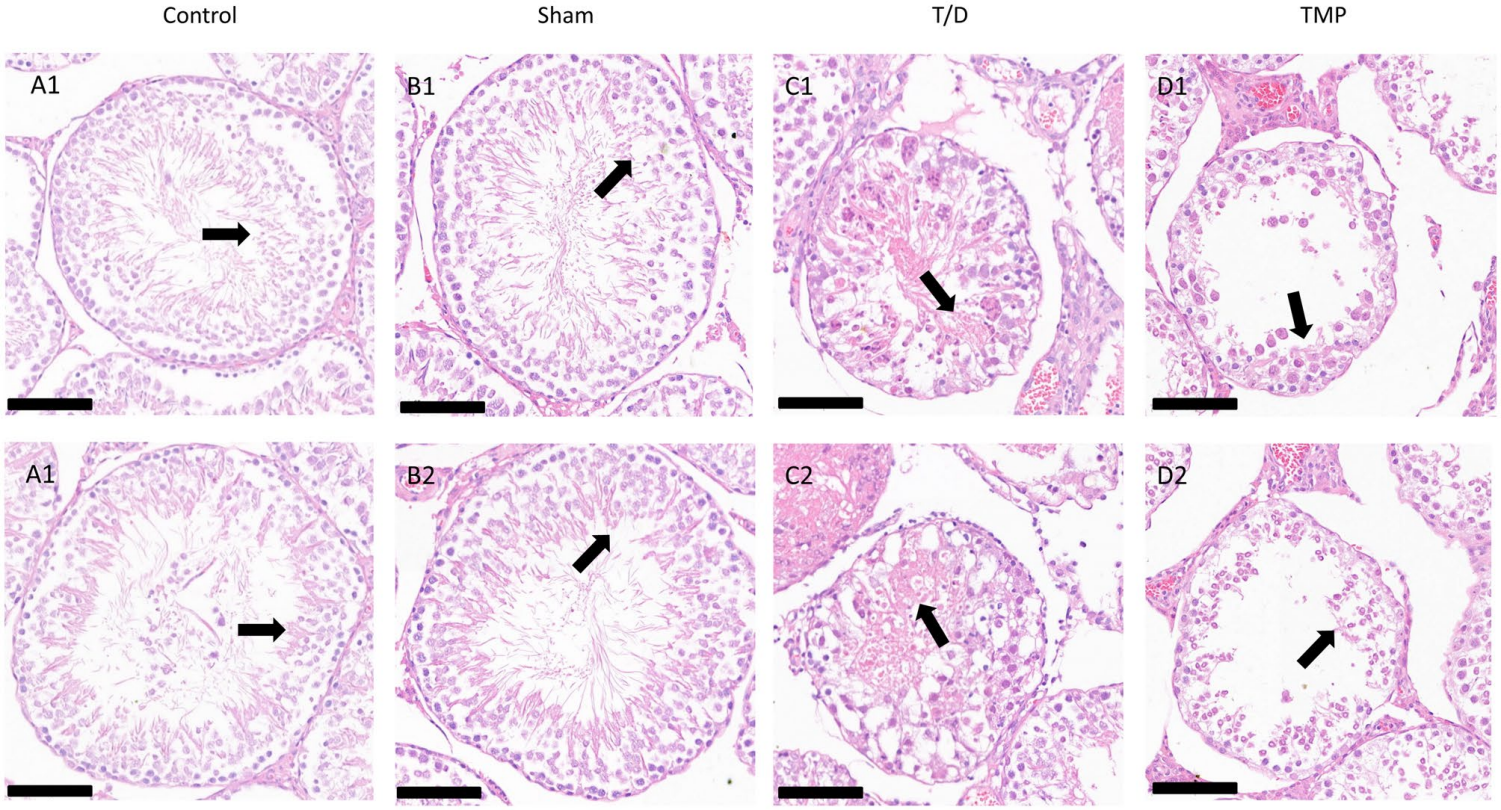
Testicular histopathology (magnification, 200x; scale bar, 100 μm): All the testicular seminiferous tubules in the Control group (**A1**, **A2**) and Sham group (**B1**, **B2**) had a normal diameter and structure; also, the spermatogenic cells in the tube wall were regularly arranged with clear layers. Of note, the quality is normal. However, in the T/D group (**C1**, **C2**), the seminiferous tubules of the testis were significantly atrophied, with incomplete structure; additionally, the spermatogenic cells on the tube wall were disorderly arranged, with unclear layers and a large number of shedding. In the TMP group (**D1**, **D2**), the seminiferous tubules were slightly atrophied, and the spermatogenic cells on the tube wall were slightly disordered with recognizable layers. A small number of spermatogenic cells fell off in the lumen, and the interstitium was slightly edema.

By examining the testicular tissue morphology of the four groups using microscopy (Fig. 2), we discovered that the T/D group had fewer cell layers in the germinal tubules of the testicular tissue (Fig. 2B1–B2) compared to the Sham group (Fig. 2B1–B2), besides, the germ cells were shed, with an irregular and edematous tissue structure. On the other hand, the germ cells in the TMP group (Fig. 2C1–C2) were neatly arranged with a clear hierarchy between cells than that of the T/D group; nonetheless, it was not as good as the normal tissue of the Sham group. The results of the Sham group and Control group were similar (Fig. 2D1–D2). Thus, Ligustrazine could significantly reduce the testicular tissue damage caused by testicular torsion.

### Comparison of testicular tissue Johnsen scores in four groups of rats

**Figure 3:**
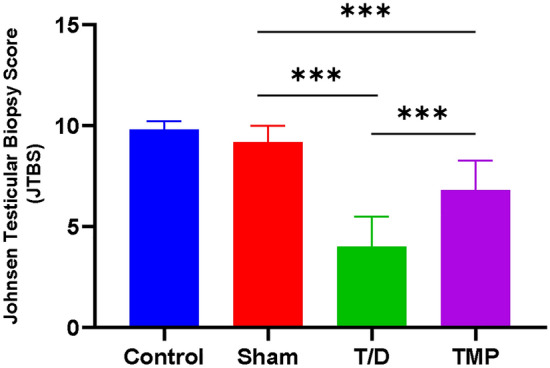
Comparison of Johnsen scores in three groups of rats; *Note* *P < 0.05; ⁎⁎*P *< 0.01; ⁎⁎⁎*P *< 0.001.

The Johnsen score for testicular tissue in the T/D group (4.00 ± 1.49) was significantly lower than that in the Sham group (9.20 ± 0.79), indicating severe damage (*P *< 0.0001). Conversely, the Johnsen score for testicular tissue in the TMP group (6.80 ± 1.48) was significantly higher than that in the T/D group (*P *= 0.0005), but lower than that in the Sham group (*P *= 0.0003). The results demonstrate a considerable improvement in testicular tissue damage post the Ligustrazine intervention. Additionally, there remains a noteworthy reduction in the Johnson score in the TMP group when compared to the Sham and Control group (Fig. 3).

### Apoptosis of germ cells

**Figure 4:**
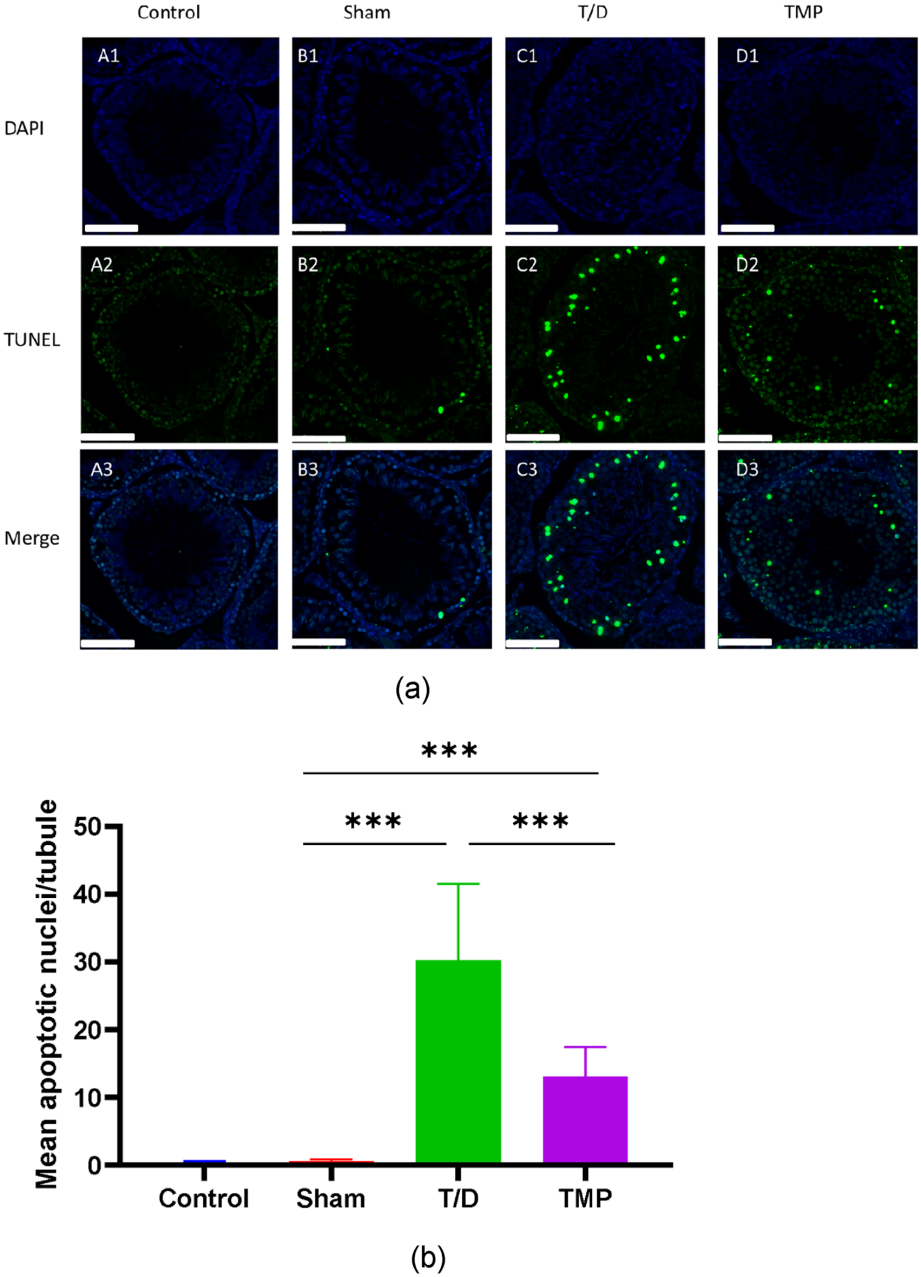
(**a**) TUNEL staining of apoptotic nuclei in the seminiferous tubules (magnification, 200x; scale bar, 100 μm). A few apoptotic nuclei were in testes of Sham group (**B1**, **B2**, **B3**) and Control group (**A1**, **A2**, **A3**). Apoptotic germ cells significantly increased in the T/D group (**C1**, **C2**, **C3**). Apoptotic nuclei in the testes of the TMP group (**D1**, **D2**, **D3**) were fewer than that in the T/D group. (**b**) Mean apoptotic nuclei/tubule. *Note* *P < 0.05; ⁎⁎*P *< 0.01; ⁎⁎⁎*P *< 0.001.

TUNEL assay confirmed the apoptosis of germ cells (Fig. 4a). Germ cell apoptosis was significantly higher in the T/D group than that in the Sham and TMP groups, and TMP group was also significantly high than sham group. Hence, ligustrazine treatment significantly reduced apoptosis compared to the T/D group.

Apoptotic index (mean apoptotic nuclei/tubule) was calculated based on the findings of TUNEL staining. The apoptotic index (mean apoptotic nuclei/tubule) of germ cells in the TMP group (13.05 ± 4.41) was significantly lower than that in the T/D group (30.23 ± 11.31) (*P *= 0.0003) and higher (*P *< 0.0001) than that in the Sham group (0.56 ± 0.29) (Fig. 4b).

## Discussion

Testicular torsion is a prevalent urological emergency that necessitates surgical intervention. The Ischemia Reperfusion Injury (IRI) after testicular detorsion decreases male fertility^1^. The current research findings documents that reactive oxygen species (ROS) is one of the primary mechanisms of IRI^19–21^. In normal physiological conditions, the body produces a certain amount of ROS essential for triggering important physiological functions, which are immediately scavenged by the *SOD* and *CAT* or other reactive oxygen species scavengers without causing damage to the body's cells. Nonetheless, in pathological conditions for instance IRI, the body produces above normal *ROS*, and since various intracellular enzymes have metabolic disorders, the excess *ROS* cannot be completely scavenged by the body, thereby causing cell damage and death or apoptosis of spermatogenic cells in the testis^22,23^. Therefore, anti-oxidant therapy is considered an effective treatment for IRI^24,25^. Chuanxiong has been clinically used in China for thousands of years as traditional Chinese medicine, and Ligustrazine, its active ingredient has been a subject of intense studies which indicate that Ligustrazine regulates anti-oxidant, anti-inflammatory, cell apoptosis, and calcium-homeostasis^1,26–28^.

Ligustrazine has antioxidant and protective properties against IRI in the heart, brain, kidney, liver, and other organs^9,10,26,29,30^. Su et al.^31^ discovered that Ligustrazine attenuates coronary microembolization-induced myocardial injury by increasing serum NO (nitric oxide) and SOD activities as well as decreasing the serum level in MDA, c-TnI (c-troponin I), and TNF-α (tumor necrosis factor-alpha). Zheng et al.^32^ reported that Ligustrazine promotes endogenous oxygen-free radical scavenging of rats with ischemia–reperfusion lung injury. This study found that Ligustrazine has an antioxidant effect on testicular tissue after detorsion. GPX, SOD and CAT activities were decreased, while MDA and ROS levels were increased in the TMP and T/D groups compared to the Sham group (*P *< 0.05). However, the TMP group showed a significant increase in the enzymatic activities of GPX, SOD, and CAT, whereas the level of MDA and ROS decreased in contrast to the T/D group (*P *< 0.05). Therefore, our study illustrates the antioxidant effect of Ligustrazine in the rat testicular torsion model.

Excess *ROS* attack and react with unsaturated fatty acids on cell membranes in lipid peroxidation, causing structural damage to biological membranes, as well as proteins and DNA damage. This induces apoptosis, therefore resulting in tissue and organ dysfunction^33–35^. Sperm cell membranes are rich in unsaturated fatty acids, and sperm have little capacity to scavenge *ROS*, making them vulnerable to *ROS* attack^36^. Lipid peroxidation occurs in sperm cell membranes after *ROS* attack, thereby decreasing cell membrane fluidity, which influences sperm capacitation and acrosome reaction^37^. As such, the production of excessive *ROS* after testicular torsion might trigger spermatogenic cell damage, testicular function loss, and reduced fertility^38^.

Notably, the follow-up treatment plan after testicular torsion-detorsion seeks to preserve fertility. We compared the testicular tissue between the TMP group and the other two groups (T/D group and Sham group). Consequently, we noted that Ligustrazine could significantly ameliorate testicular tissue damage caused by torsion. Moreover, the testis tissue in the TMP group was significantly better than that in the T/D group and worse than in the sham group. Moreover, the Johnsen score of testicular tissue in the TMP group (6.80 ± 1.48) was significantly higher (*P *< 0.05) than the T/D group. These results illustrate a significant improvement in testicular tissue damage with Ligustrazine treatment after testicular torsion-detorsion in rats.

Our study revealed that Ligustrazine attenuates apoptosis in seminiferous tubules induced by IRI. In addition, TUNEL findings showed fewer apoptotic nuclei in the testes of the TMP group than that in the T/D group. Therefore, Ligustrazine decreases apoptotic germ cells. Of note, apoptosis has been commonly observed in the testis ischemia–reperfusion injury animal model, and germ cell apoptosis was maximal at 24 h after detorsion^39–41^. Elsewhere, a study found that the testicular torsion and detorsion cause severe histological damage at spermatogenesis and spermiogenesis levels; the testicular torsion-detorsion improved the mRNA damage and DNA fragmentation^42^. Furthermore, Aslan Kosar asserted that testicular torsion–detorsion injury caused cell damage and germ cell apoptosis that positively correlates with cleavage of Poly (ADP-Ribose) Polymerase^39^. Therefore, combining our results with related studies, we conclude that Ligustrazine reduces testicular apoptosis by alleviating oxidative stress, but the underlying molecular mechanisms still need to be further explored.

## Conclusion

The results of our study indicate that Ligustrazine reduces ischemia–reperfusion injury (IRI) caused by testicular torsion-detorsion through a decrease in oxidative stress and inhibition of apoptosis. These findings suggest the therapeutic potential of Ligustrazine for the clinical treatment of testicular IRI. However, further research is required to investigate the molecular mechanisms involved in the inhibition of apoptosis by Ligustrazine. Additionally, more clinical studies are necessary to corroborate our findings.

## Data Availability

Data are available upon reasonable request from the corresponding author.
